# THE RELATIONSHIP BETWEEN COGNITION, FUNCTIONAL ABILITIES, AND THE LATENT DEMENTIA PHENOTYPE AMONG CENTENARIANS

**DOI:** 10.1093/geroni/igz038.3498

**Published:** 2019-11-08

**Authors:** Jonathan Sober, John L Woodard, L Stephen Miller, Adam Davey, Peter Martin, Leonard Poon

**Affiliations:** 1 Wayne State University, Detroit, Michigan, United States; 2 University of Georgia, Athens, Georgia, United States; 3 University of Delaware, Newark, Delaware, United States; 4 Iowa State University, Ames, Iowa, United States

## Abstract

Adequate assessment of cognitive abilities and functional capacity is essential for a diagnosis of dementia. However, cognition is only moderately related to functional status, and this relationship is poorly understood among centenarians, a group of older adults with high risk for dementia. A bifactor structural equation model can be used to delineate the variance attributed to dementia-specific related cognitive changes (i.e., the latent variable delta) and the variance due to general intelligence (i.e., g’). This study aimed to determine the validity of delta as a marker of cognitive decline among centenarians. It was hypothesized that delta was correlated with cognitive status, functional abilities and, dementia severity. Overall, 244 community dwelling centenarians (Mage = 100.58, 84.8% female) were recruited through the Georgia Centenarian Study, a population-based study of octogenarians and centenarians from northern Georgia. Older adults were administered measures of cognition and a self-report measure of functional abilities. Latent variable scores (i.e., g’ and delta) were modeled and correlated with standard global cognitive screening measures (i.e., MMSE) and measures of dementia severity. Results indicate that delta was significantly correlated with functional ability and cognitive abilities. Consistent with our hypotheses, delta was also significantly related to dementia severity. Overall, estimates of the latent dementia phenotype, delta, were significantly related to cognitive and functional abilities among centenarians, providing validation of delta as a useful index of dementia severity.

